# Public Engagement Provides First Insights on Po Plain Ant Communities and Reveals the Ubiquity of the Cryptic Species *Tetramorium immigrans* (Hymenoptera, Formicidae)

**DOI:** 10.3390/insects11100678

**Published:** 2020-10-07

**Authors:** Cristina Castracani, Fiorenza Augusta Spotti, Enrico Schifani, Daniele Giannetti, Martina Ghizzoni, Donato Antonio Grasso, Alessandra Mori

**Affiliations:** Department of Chemistry, Life Sciences & Environmental Sustainability, University of Parma, Parco Area delle Scienze, 11/a, 43124 Parma, Italy; enrico.schifani@unipr.it (E.S.); daniele.giannetti@unipr.it (D.G.); martina.ghizzoni@unipr.it (M.G.); donato.grasso@unipr.it (D.A.G.); alessandra.mori@unipr.it (A.M.)

**Keywords:** community science, citizen science, BioBlitz, School of Ants, ant biodiversity, exotic species

## Abstract

**Simple Summary:**

Public involvement in biodiversity research in the form of Citizen Science is a powerful tool to improve our understanding of the natural world, and it is especially suitable for the study of heavily populated environments. Ants’ ubiquity and diversity, their role as ecological bioindicators, and the fact that most species can easily be sampled makes them ideal candidates for this kind of studies. In the framework of the international School of Ants citizen science project, we joined the “BioBlitz Lombardia” in which citizens are invited to collect biodiversity data on several parks from Lombardy (Po Plain, Italy). As a result, we recorded 30 ant species and obtained a first characterization of the region’s ant assemblages. We studied their patterns of variation in relation with the ecological difference between the studies sites, which ranged from urban to subalpine areas. In addition, we detected the presence of a cryptic species (*Tetramorium immigrans*) whose distribution and identity were only recently clarified. It likely represents an under-recorded introduced species in the region. Advantages and critical aspects of using CS methodology for the study of biodiversity are discussed in light of our experience.

**Abstract:**

Ants are considered a useful model for biodiversity monitoring and several of their characteristics make them promising for citizen science (CS) projects. Involving a wide range of public figures into collecting valuable data on the effect of human impact on ant biodiversity, the School of Ants (SoA) project represents one of the very few attempts to explore the potential of these insects in CS. Through the collaboration with the “BioBlitz Lombardia” project, we tested the SoA protocol on 12 Northern Italy parks, ranging from urban green to subalpine protected sites. As a result, we obtained some of the very first quantitative data characterizing the ants of this region, recording 30 species and highlighting some interesting ecological patterns. These data revealed the ubiquitous presence of the recently taxonomically defined cryptic species *Tetramorium immigrans,* which appears to be probably introduced in the region. We also discuss advantages and criticisms encountered applying the SoA protocol, originally intended for schools, to new categories of volunteers, from BioBlitz participants to park operators, suggesting best practices based on our experience.

## 1. Introduction

Public engagement in scientific projects has been known since the beginning of the 20th century [1], but its value has been only recently recognized by the scientific community that names citizen science (CS) this collaboration between scientists and citizens [1,2,3,4]. Citizen science provides many advantages to scientists, allowing them to gather a high amount of data with reduced time and economic costs, and, at the same time, it enhances citizen awareness and education over scientific practices and topics [2,5,6,7]. 

Recently, the expression “community science” was proposed as a more inclusive replacement of the widely used expression “citizen science,” in the attempt to highlight that all volunteers can participate to scientific projects, regardless of their origin and background [8,9]. Nevertheless, following the UNESCO Recommendation on Open Science [10], Wehn and colleagues [11] maintained the term “citizen science,” making it a pillar of Open Science as it promotes social inclusion, scientific citizenship and sustainability. Moreover, the expression “community science” is often used in relation to projects that are led by the community itself to address a collective issue, sometimes even without the help of experts [12]. As a consequence, in this paper, we decided to keep the consolidated term “citizen science” to enhance clarity throughout the text and avoid confusion in relation to literature we referred to. However, we intend the word “citizen” with the broad sense of “nonspecialist participant.”

CS projects can effectively contribute to attain most of the Sustainable Development Goals (SDGs) scheduled by United Nations in the 2030 Agenda for sustainable development [13,14,15]. In fact, in accordance with SDG n. 15 “Life on land,” many CS projects aim at monitoring biodiversity for conservation purposes over regional to continental extents and decadal time scales [7,15,16]. Biodiversity preservation appears as urgent as ever due to the ongoing environmental crisis [17], therefore, CS offers key opportunities to collect rapidly fine-grain data on a broad spatial scale and to raise public awareness on this issue [4,5,7,16,18,19,20,21].

A critical point for monitoring biodiversity in CS projects is selecting a target group easy to observe and representative of its ecosystem. For this purpose, ants (Hymenoptera, Formicidae) can be a suitable model because they are abundant and widespread in most terrestrial ecosystems and easy to detect and collect also for nonexperts. Moreover, ants belong to an extremely diverse family of insects and, thus, they are a good model to explain the meaning of the term “biodiversity” to citizens. Finally, ants are often keystone species in their ecosystems and they can respond quickly to environmental changes, so they are successfully used as bioindicators in various contexts [22,23,24,25,26,27,28,29].

Due to the growth of urbanization worldwide, biodiversity conservation in the cities has become a major concern [30,31,32] and it is part of the SDG n. 11 “Sustainable cities and communities” [13]. From this perspective, monitoring ant communities can be critical to assess the impact of human activities on urban ecosystems, but few data are currently available, mainly referring to the American and Australian continents [33,34,35,36,37,38]. In Italy, the role of ants as bioindicator is still poorly studied and the few researches were conducted in protected natural areas [39,40,41,42,43,44].

First launched in the United States [45,46] and then spread to other countries [47,48,49], the citizen science project “School of Ants” (SoA) aims to fill this knowledge gap, studying urban ant biodiversity through large public involvement. In Italy, the project, led by the Myrmecology Lab (University of Parma—UNIPR), has further developed around the need of counteracting the scarcity of practical scientific experiences within Italian schools and it has now flown into the project “School of Ants: a scuola con le formiche” (SoA: learning with the ants). This lack of an inquiry-based science education is likely to be the main cause of students’ low interest for scientific subjects and, subsequently, for scientific professions [50,51]. In this context, the SoA project offers to students and teachers a hands-on science experience with the ongoing support of a team of researchers. Moreover, the SoA project provides participants with a very simple sampling protocol to address the data quality issue, whereas researchers perform the critical step of species identification. The last step is the production of feedback for participants: results are shown and directly discussed with volunteers or through emails, the official website, and the social networks. After a pilot phase focused on the ant fauna of the urban area of Parma (Emilia-Romagna, Northern Italy) (see box “School of Ants goes global: International module of SoA in Parma, Italy” in [46]), the project eventually spread nationwide thanks to the collaboration with the Science Museum of Trento [52]. While the initial results had depicted the commonness of the ant *Tetramorium caespitum* (Linnaeus, 1758) [46], new research questions arose from recent taxonomic advancements. Since the split of the traditional concept of *T. caespitum* into a complex of several species characterized by different ecology and natural history [53,54], the distribution of the complex could now be studied on a nationwide scale thanks to the SoA greater geographic reach.

In 2018, the SoA met the “BioBlitz Lombardia” (BBL), a project managed by a network of parks [55] and the Lombardy Region [56]. Traditional BioBlitz events aim at discovering and recording the presence of as many species of plants, animals, and fungi as possible within a set location, over a defined period (usually 24 h) and they are run by professional naturalists with public participation [57,58,59]. The BBL project does not fully comply with the traditional BioBlitz definition because it was not held continuously during 24 h, but during different time slots along a weekend, depending on each park’s choice. However, it was a collaborative monitoring event where nonspecialist volunteers were guided by experts to discover local biodiversity and to upload the records on a global dataset platform [60]. On one hand, the BBL was looking for a taxonomic group that could be found in all the parks in order to compare data along different parks and to give an additional cohesive element to the network. On the other hand, the SoA was looking for new categories of volunteers to recruit in order to collect more data from new sampling sites. As a result, sampling through the SoA protocol was conducted in the most anthropogenic areas of some parks adhering to the BBL 2018 to keep the SoA focus on urbanized spots. According to CS best practices [61], participants received feedback on data they collected, as the checklist of the ant species and information on their abundance and distribution.

As data on ant communities in the study areas were out of date and incomplete [62], this work aimed mainly to: (i) test the effectiveness of the SoA project in involving a new category of volunteers as the people attending the biodiversity BioBlitzes; (ii) gather data on species abundance in order to provide an updated, even if not comprehensive, checklist of the ant species and to compare the ant communities of different parks; (iii) obtain new insights on the distribution of *T. caespitum* complex species in Italy.

## 2. Materials and Methods 

### 2.1. Project Development

The project was first presented on February 2018 during the workshop “BioBlitz Lombardia: a review of the past editions and future plans” organized by Lombardy Region, A.R.E.A. Parchi, Oglio Sud Park, and Le Bine Regional Reserve. Addressed to the representatives of the parks joining the A.R.E.A. Parchi network, the workshop was the first meeting between the researchers of the SoA project and the organizers of the BBL. In this occasion, goals of both SoA and BBL were presented and a first draft of the joint project “SoA at BioBlitz Lombardia 2018” was attained.

In the following weeks, a participation list from parks of the A.R.E.A. Parchi network was compiled and Parco Nord Milano organized a training meeting at the end of March 2018 (see Table 1; Table S1). During the meeting, the park representatives were trained by the UNIPR researchers on the SoA sampling protocol with a specific attention to the sampling sites. Le Bine Regional Reserve was used as a study model and a simulation of the sampling sites selection was proposed and analyzed. Parco Nord Milano area was then used for a demonstrative sampling: all the participants were allowed to use one kit and they were also trained for the use of the iNaturalist App. Finally, each participant received the material for setting a SoA point during the BBL 2018: 10 ant sampling kits, a set of brochures on SoA project, and a set of brochures about the project “SoA at BBL 2018” for sharing with BioBlitz participants. In order to keep update the participants at the meeting, a mailing list was created and a cloud was used for sharing documents.

The BBL 2018 edition took place on 19 and 20 May 2018 and all the ants were sampled in those 2 days. During the months of June and July, all the samples were sent to the Myrmecology Lab.

From July to November 2018, all the ants were identified and each park received a set of results that they could share with the BioBlitz volunteers: a check list of the sampled species, an identity card for each species, a dataset recording the number of baits containing the species collected in the park (species frequencies), and a set of pictures of the specimen to be uploaded in the iNaturalist platform.

In December 2018, a final workshop was organized by Lombardy Region and A.R.E.A. Parchi in order to show and discuss the achievements in terms of both scientific results and good practices to suggest.

### 2.2. Sampling

**Figure 1:**
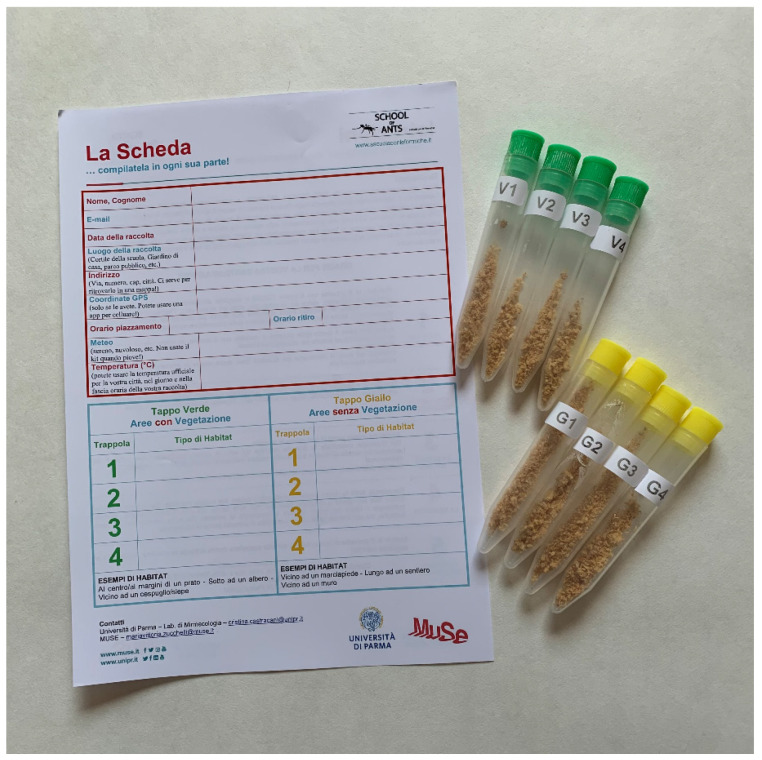
The ant collection kit: the data collection sheet (on the left) and the 8 baits (on the right).

During BBL 2018, each park participating to the SoA project set up a SoA point where park operators explained to visitors the aims of the project, recruited volunteers, and helped them to apply the protocol for ant collection properly. The Myrmecology Lab provided each park with 10 collection kits and with the instructions on how to use them. Park operators were invited to choose one or two sampling sites in anthropogenic areas within the park (e.g., near the visitor center or other buildings), and to use at least 5 kits at the same site, that is a place defined by the same geographical coordinates. The sampling protocol was the same described as “modified SoA protocol” in Lucky et al. 2014 (see box “School of Ants goes global: International module of SoA in Parma, Italy”). A kit contained 8 baits consisting of 12-mL falcon tubes (4 with green caps and 4 with yellow caps) filled with 4 mL of cookie crumbs for attracting ants and a data collection sheet to fill with some collector personal data and environmental/weather information (Figure 1). Volunteers were asked for using a kit as follows: 1. opening and placing the 4 green-capped baits in vegetated areas (e.g., on the grass or at the base of a tree); 2. opening and placing the 4 yellow capped baits in areas without vegetation (e.g., on concrete surfaces or paved areas); 3. waiting 1 hour and, in the meanwhile, filling the data collection sheet; 4. collecting all the baits, capping them, and returning them to park operators together with the compiled data collection sheet. Twelve parks were involved in the SoA Project and 14 sites were sampled during BBL 2018 (Table S1).

### 2.3. Species Identification

Specimens were examined under a ZEISS Stemi 508 stereoscopic microscope (5–200× magnification range) with the asylum of an Axiocam Erc 5s and ZEISS ZEN core software used to take morphometric measurements. They were identified according to the following keys [63,64,65]. *Tetramorium* species belonging to the *caespitum* complex were identified according to the key provided by Seifert [64]: morphometric measurements were taken up to 2 workers per bait, assuming multiple specimens belonged to the same colony. All specimens were finally stored in the ant collection of the Myrmecology Lab.

### 2.4. Statistical Analyses

Statistical analyses were performed using the software R 3.5.2 and RStudio 1.1.463 [66]. Analyses were carried out on two datasets: Bulleted lists look like this:

- Full Dataset (FDataset)—This dataset contains data (species richness and abundance) from all the kits employed during BBL
- Standardized Dataset (StdDataset)—This dataset was created as a subset of the previous one with the aim of standardizing the sampling effort among different sites. We excluded data from sites in which less than 4 kits (32 baits) were employed. Moreover, for all the sites where more than 6 kits were used, we randomly selected only 6 kits (48 baits) per site.

FDataset was used for computing species accumulation curves of each site. The curves were plotted by considering the presence/absence of the ant species in each bait and using the specaccum () function of the Vegan R package [67]. 

Data from StdDataset were used to compare sites through the Nonmetric Multidimensional Scaling (NMDS). The average number of baits in which the species were found in each site was used in the analysis, and the metaMDS () and envfit () functions of Vegan R package were used for computation [62]. For each site, the StdDataset was used for calculating species richness and the following biodiversity indices: Simpson’s Diversity Index (1-D), Shannon Diversity Index (H’), and Species Equitability Index (EH) [68,69]. All indices were calculated based on frequencies (i.e., the number of baits in which each species was found). The same dataset was then used for calculating the Sørensen–Dice Similarity Index expressed as the mean value among all the possible comparisons with every other site [70,71]. 

## 3. Results

Data from the FDataset (768 baits from 14 sites) recorded the presence of 30 ant species belonging to 3 subfamilies: Dolichoderinae (1 sp.), Formicinae (12 spp.), and Myrmicinae (17 spp.) (Table 2 and Table S2). Eleven of these species were new to the Italian SoA project. The most speciose genera were *Lasius* Fabricius, 1804 (5 spp.), and *Temnothorax* Mayr, 1861 (5 spp.), followed by *Camponotus* Mayr, 1861 (4 spp.), and *Myrmica* Latreille, 1804 (4 spp.). Concerning site biodiversity, we recorded from 5 to 14 species per site (Table S2).

**Figure 2:**
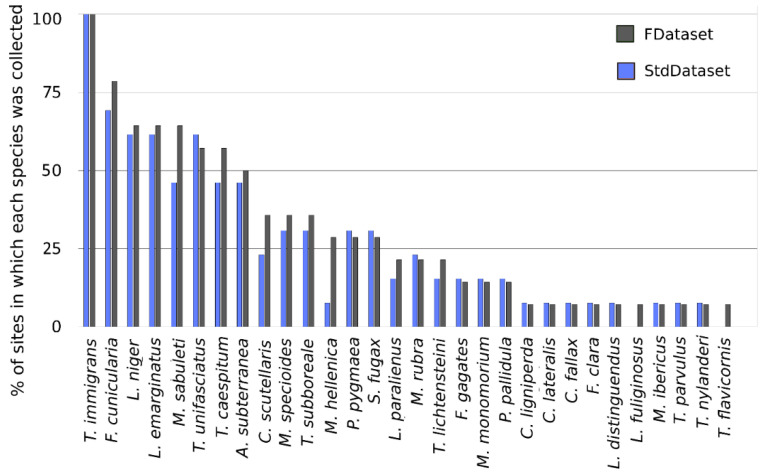
Distribution of ant species along the sampling sites. In gray, data from the FDataset (N = 14) and in blue, data from the StdDataset (N = 13). Species are listed according a decreasing gradient calculated from the FDataset.

The most widespread species was *T. immigrans*, collected in all 14 sites, whereas the following species were recorded in 1 site only: *C. fallax*, *C. lateralis*, *C. ligniperda*, *F. clara*, *L. distinguendus*, *L. fuliginosus*, *M. ibericus*, *T. flavicornis,* and *P. pallidula* (Figure 2—blue bars and Table S2).

**Figure 3:**
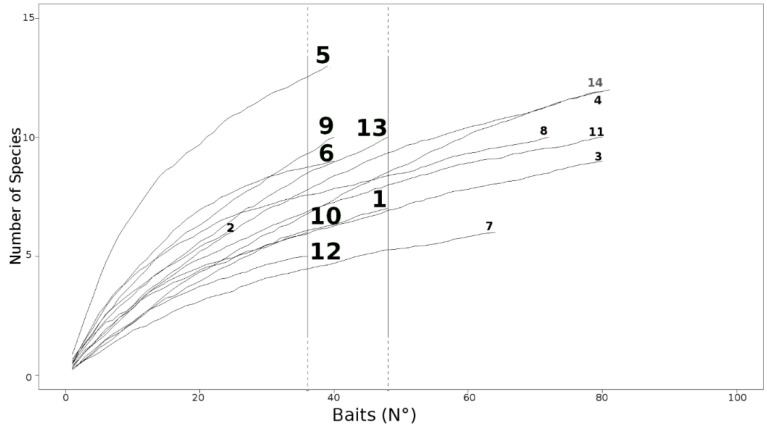
Species accumulation curves of each sites, calculated using the FDataset. Vertical lines show the range of baits (36–48) chosen for creating the StdDataset. The numbers refer to sites according to the list in Table 1.

The species accumulation curves showed that no complete plateau was reached in any of the sites, although in most sites, the curves reduced their increasing trend around 40 baits (Figure 3). Therefore, in the StdDataset, all data from site 2 as well as some data from sites 3, 4, 7, 8, 11, and 14 were removed.

**Figure 4:**
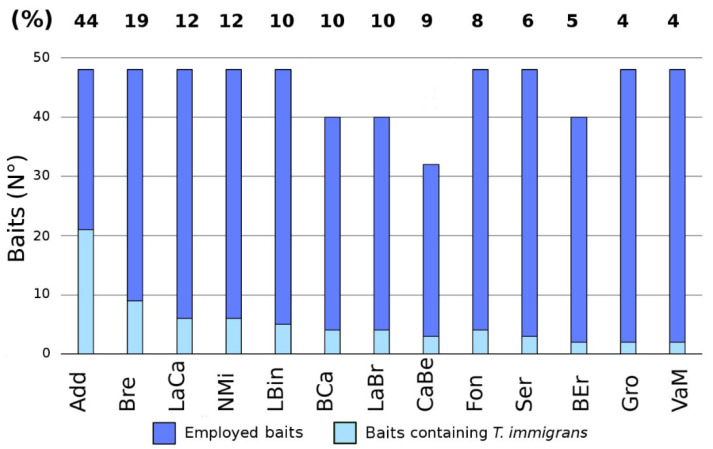
Occurrence of *Tetramorium immigrans* in each site (StdDataset). The percentage (%) of baits containing *T. immigrans* is indicated on top of each bar.

Data from the StdDataset (568 baits from 13 sites) recorded the presence of 28 ant species. Concerning site biodiversity, we recorded from 5 to 14 species per site. *Camponotus*, *Lasius*, *Myrmica,* and *Temnothorax* were still the richest genera, while the rarest species were the same of the FDataset except for the absence of *L. fuliginosus* and *T. flavicornis* (Figure 2; Table S2). *Tetramorium immigrans* was again present in all 13 sampling sites, occurring on average in 12% of the baits per site (Figure 4 and Table S2).

**Figure 5:**
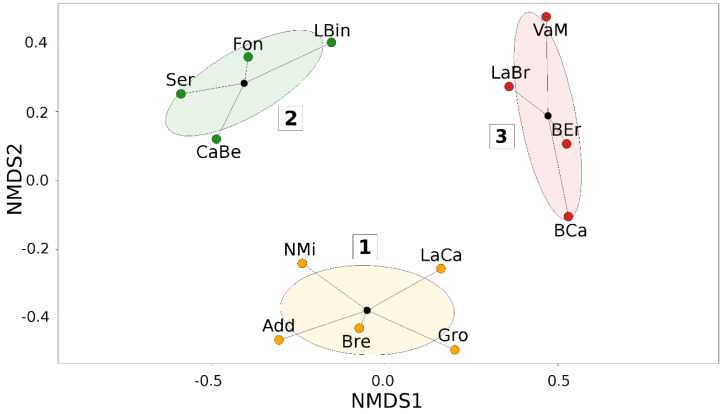
Nonlinear Multidimensional Scaling of the sites according to the average number of baits in which each species was found (StdDataset). Clusters are marked with different colors. For each cluster, ellipses show the standard error and black dots represent the centroids.

**Figure 6:**
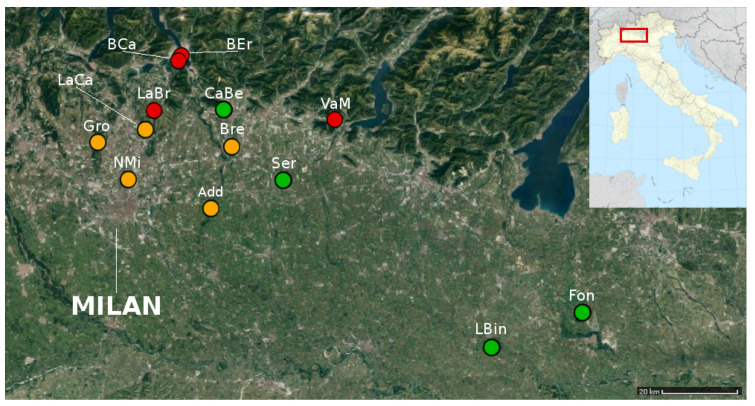
Spatial distribution of sampling sites (StdDataset). Each dot represents a site. Colors correspond to the clusters found in the NMDS analysis: cluster 1—orange, cluster 2—green, and cluster 3—red. Map: satellite image from Google Maps retrieved on 1 March 2020 and modified by authors.

The NMDS analysis on sampling sites showed the presence of 3 clusters (stress value = 0.18, R^2^ = 0.815, *p* = 0.002) (Figure 5). Sites were then located in a map and clusters were shown with different colors (Figure 6).

Species richness, biodiversity indices, and Sorensen index are reported in Table 3. NMDS clusters differed in species richness, biodiversity indices, and in their most frequently collected species. There is an increasing gradient from cluster 1 to cluster 3 for both species’ richness and all the biodiversity indices. Similarity index values were similar among sites ranging from 0.35 to 0.50 (Table 3 and Table S3).

## 4. Discussion

The “School of Ants” and the “BioBlitz Lombardia” are two Citizen Science projects currently operating in Italy. The SoA project aims to analyze the distribution of Italian ant fauna in urban environments for collecting information about the effects of human impact on urban biodiversity, and acting, at the same time, as a tool to introduce the volunteers to scientific research thorough a hand-on experience. The main goal of BBL is promoting the biodiversity of the protected areas in Lombardy through the active participations of volunteers that, helping in recording data, can understand and enhance the value of biodiversity and the importance of containing its lost. The collaboration between the two projects during BBL 2018 was successful in many aspects. The SoA project was opened to new type of volunteers contributing to expand the number of sampling sites and consequently, the database of the identified species. The checklist of urban ants sampled through the SoA project in Italy increased remarkably, helping to fill the knowledge gap on Italian ant species distribution that is one of the main goals of the project. Networking is a very strong value of the BBL: in Italy, this is one of the few examples where a huge effort in coordinating activities of very different participants has been done to support and promote a common vision. BBL found in ants a suitable zoological taxon to be transversally investigated and so improving cohesion among the several protected areas working in the project.

Applying the SoA protocol proved to be remarkably cost-effective for achieving a first quantitative list of ant species in the sampled sites. We were able to detect a relatively high number of different taxa and to provide some key information over the ant communities of the investigated sites, although the sampling effort was limited to just 2 days and to a single methodology (which is obviously unlikely to detect strictly arboreal, endogean, or insectivorous ants).

However, a nonhomogeneous sampling effort forced us to reduce the database with an unavoidable loss of information. This aspect was probably underestimated during the training workshop for park executives led by the SoA researchers. From experiences with school teachers, a particular attention was devoted to the sampling protocol and to the identification of sampling sites, but concepts related to sampling effort were overlooked. Our experience suggests that whenever new figures between researchers and volunteers, such as park executives, are involved, their training is a crucial step and additional efforts need to be done in order to test their effective understanding of the goals and the methods of the project.

Even though the aim of this research was far from obtaining an exhaustive checklist of the investigated areas, the resulting data represent a valuable contribute to the general knowledge of the region: quantitative surveys on the Po Plain ant fauna are still lacking, and existing faunistic data are scattered across an outdated literature (see [62]). Biogeographically, according to the available species distribution information [64,72,73], Eurasian or European species characterized by considerably wide distribution ranges appeared to be prevalent: roughly one-third of the species (e.g., *F. clara*, *F. cunicularia*, *L. niger*, *L. fuliginosus*, *M. rubra*, *M. specioides*) could be referred to the ASE chorotype and another third (e.g., *C. ligniperda*, *M. sabuleti*, *L. paralienus*, *T. caespitum*, *T. nylanderi*, *T. parvulus*) to the EUR chorotype [74]. The rest of the species are characterized by smaller distribution ranges, gravitating around Southern Europe (*F. gagates*, *M. ibericus*, *M. monomorium*, *T. lichtensteini*), South-Western Europe (*P. pallidula*), South-Eastern Europe (*M. hellenica*, *T. flavicornis*), the Western-Mediterranean (*C. scutellaris*), or were circum-Mediterranean (*C. lateralis*). However, no endemic taxa were found. The overall prevalence of a Central-European fauna, mixed with wide-range South-European species and only limited Mediterranean elements matches our expectation from a Southern-European region characterized by continental climatic conditions and biogeographically equally related to the Eastern and Western Mediterranean sectors (see [75]). Finally, all detected *Myrmica* spp. represent potential hosts for endangered *Phengaris* Doherty, 1891 lycaenid butterflies [76,77], that are present in Northern Italy and were recorded in some of the investigated parks (e.g., [78,79]).

Although sampling in the most anthropogenic spots of each park, as the SoA protocol demanded, could lead to a strong homogenization of data, the level of detail we achieved was sufficient to highlight a quite clear separation of the sampling sites into three groups. In the first group, *L. niger* and *T. immigrans* were the most abundant species. Both are disturbance-tolerant taxa particularly well known to adapt successfully to urban environments [64] and, interestingly, this group is made of lowland sites geographically closely related to the large Milan metropolitan area. In the second group, *Myrmica* spp. and *M. monomorium*, ecologically linked to meadows, were prevalent. Sites of the second group are averagely more distant from the metropolitan area and closer to areas where agricultural land use prevails. Finally, in the third group, the most abundant species were *L. emarginatus* and *A. subterranea*, usually encountered in broad-leaved woodlands. Sites of this group are located at the foothills of the pre-Alps, in which more natural conditions occur. A more detailed investigation on the relationship between these ecological conditions and the composition of investigated ant communities is beyond the scope of the present paper. However, these indications once again highlight the potential of ant communities as bioindicators, still little explored in Italy [39,40,41,42,43,44]. 

Another interesting result was the evidence of *T. immigrans* ubiquity: although only prevalent in the sites closer to the metropolitan area, it was also the only species found in all of the sampling sites. The *T. caespitum* complex, to which *T. immigrans* belongs, was until recently considered to be represented by a single species in Europe. However, it is now considered a cryptic complex of 10 different taxa in the region whose distribution data are often scarce [53,54,80]. Among the species of this complex, only *T. immigrans* is known to possess a notable invasive potential, as it has colonized large areas of North America, gaining since the 19th century the nickname of “pavement” ant due to its extreme success in urban environment (e.g., [81,82,83,84]). *Tetramorium immigrans* is certainly native in the Anatolia and Caucasus regions, where it shows a comparatively high haplotype diversity and inhabits natural mountain habitats [53,85]. On the other hand, it appears to be exotic at least in some regions of Europe, where its haplotype diversity is much lower (comparable to North America, see [53]) and, as most introduced ant species (see [86]), it is mainly concentrated in urbanized areas [49,53,87,88,89,90,91,92,93,94]. For example, this situation led to the idea that *T. immigrans* is an introduced species in both Denmark and Greece [53,90]. In these cases, unlike in North America, *T. immigrans* does not enjoy the easy taxonomic recognition that the numerous other introduced *Tetramorium* spp. have in Europe; instead, due to the existence of several native cryptic species, it can be classified as a cryptic alien [95]. This whole figure appears somewhat similar to that of *L. neglectus* Van Loon, Boomsma, and Andrasfalvy, 1990, introduced from Anatolia to Europe (from Greece West to Iberia) about 20 years before being taxonomically recognized [64].

In Italy, the situation appears very similar. In Sicily, *T. immigrans* corresponds to the species about 20 years ago described as a recent colonizer, large species exclusively thriving in severely altered habitats, and named as “*Tetramorium caespitum*,” living spatially segregated from the other species of the complex, which are exclusively montane on the island [89,94,96]. When the cryptic complex was taxonomically revised, species identification of the specimens from about 95 Italian localities was provided [53]. Across the six species existing in Italy, the number of urban or near-urban localities for each taxon was about 0% in the case of *T. alpestre* Steiner, Schlick-Steiner, and Seifert, 2010 (N = 22), *T. caespitum* (N = 46), *T. impurum* (Foerster, 1850) (N = 4), and *T. indocile* Santschi, 1927 (N = 3) and 33% for the Italian endemic *T. fusciclava* Consani and Zangheri, 1952 (N = 6), whereas 100% for *T. immigrans* (N = 14) (Wagner et al. 2017). It is remarkable that about 80% of the sampling sites reported by Wagner and colleagues [53] was in natural or seminatural environments and *T. immigrans* was never collected there. To better understand the status of *T. immigrans* in Italy and Europe, more detailed phylogeographic data (see also [84]) and even greater sampling efforts in natural habitats could be meaningful. However, there is currently no evidence suggesting to consider *T. immigrans* as native to the Po Plain or other Italian regions. Eventual proofs of a quick-post glacial expansion to Europe (justifying the genetic pattern shown in [53]) and of the presence of significant populations in undisturbed habitats of the country would be required to support the latter hypothesis. Until then, we suggest to consider *T. immigrans* as probably introduced in Italy similarly to the interpretations formulated in other European countries.

In the Po Plain, our data seem to testify a very different situation from Southern Italy, and possibly, quite similar to Southern France: *T. immigrans* distribution admixes with that of *T. caespitum*, forming a continuum across an ecological gradient. In France, this situation led to significant hybridization and introgression rates in the contact zone, which may seriously increase identification errors [97]. Moreover, it may suggest that strict proximity between the two species is a recent situation as stronger reproductive barriers would otherwise be expected. Future investigations in Italy could try to test for the existence of similar patterns. Moreover, the present data suggest that *T. immigrans* may be extremely common across Italian urban areas, despite being unnoticed until very recently. This result was only possible thanks to a tight collaboration between citizens and researchers, in which the role of citizens was fundamental to collect a large and valuable number of specimens in a relatively small period of time and the role of researchers was crucial due to the difficult procedures required for species identification.

## 5. Conclusions

Public engagement in biodiversity monitoring can be very useful to track the spread of exotic species, as already demonstrated in few other cases in Italy [98,99,100,101,102,103,104]. Current information over the distribution of the probably exotic *T. immigrans* in Italy remains scarce [62], but our data suggest that it is very common and widespread in disturbed habitats. Moreover, through a vast public involvement, the SoA protocol can be a powerful tool to track the distribution of this species across the country, allowing to quickly gather a significant amount of data from wide geographic areas and urban environments in particular. Since the latter are the most affected by the increasing presence of new exotic ant species [86], the SoA project may also provide more effective early-detection capabilities for the future. The possibilities offered by the use of ants for urban ecological surveys and biodiversity monitoring of disturbed sites appear still largely unchecked, but highly promising.

## Figures and Tables

**Table 1:** List of the sampling sites. The last column shows the total number of collected kits for each site.

Site Name	Tag	Latitude	Longitude	Baits
1. Adda Nord Park	Add	45°27’49” N	9°28’33” E	48
2. Campo dei Fiori Park	Fiori	45°53’10’’ N	8°42’28’’ E	24
3. Serio Park	Ser	45°31’35” N	9°43’51” E	80
4. Groane Park	Gro	45°37’14” N	9°5’36” E	80
5. Monte Barro Park, Loc. Camporeso	BCa	45°49’28” N	9°22’11” E	40
6. Monte Barro Park, Loc. Eremo	BEr	45°49’57” N	9°22’45” E	40
7. Nord Milano Park	NMi	45°32’16” N	9°12’41” E	64
8. Oglio Sud Park, Le Bine Reserve	LBin	45° 8’17” N	10°26’9” E	72
9. Valle Lambro Park, Loc. Brianza	LaBr	45°41’58” N	9°17’13” E	40
10. Valle Lambro Park, Loc. Villa Campello	LaCa	45°39’22” N	9°15’41” E	32
11. Basso Brembo Park	Bre	45°37’12” N	9°33’50” E	80
12. PLIS Monte Canto e Bedesco	CaBe	45°42’1” N	9°31’45” E	32
13. Bosco Fontana Natural Reserve	Fon	45°13’3” N	10°45’10” E	48
14. Valpredina-Misma Natural Reserve	VaM	45°43’3” N	9°48’55” E	80

**Table 2:** Checklist of the ant species identified during BioBlitz Lombardia (BBL) 2018.

Subfamily	Dolichoderinae	Formicinae	Myrmicinae
**Species**	*Tapinoma subboreale* Seifert, 2012	*Camponotus fallax* (Nylander, 1856) *Camponotus lateralis* (Olivier, 1792) *Camponotus ligniperda* (Latreille, 1802) *Formica clara* Forel, 1886 *Formica cunicularia* Latreille, 1798 *Formica gagates* Latreille, 1798 *Lasius distinguendus* (Emery, 1916) *Lasius emarginatus* (Olivier, 1792) *Lasius fuliginosus* (Latreille, 1798) *Lasius niger* (Linnaeus, 1758) *Lasius paralienus* Seifert, 1992 *Plagiolepis pygmaea* (Latreille, 1798)	*Aphaenogaster subterranea* (Latreille, 1798) *Crematogaster scutellaris* (Olivier, 1792) *Messor ibericus* Santschi, 1931 *Monomorium monomorium* Bolton, 1987 *Myrmica hellenica* Finzi, 1926 *Myrmica rubra* (Linnaeus, 1758) *Myrmica sabuleti* Meinert, 1861 *Myrmica specioides* Bondroit, 1918 *Pheidole pallidula* (Nylander, 1849) *Solenopsis fugax* (Latreille, 1798) *Temnothorax flavicornis* (Emery, 1870) *Temnothorax lichtensteini* (Bondroit, 1918) *Temnothorax nylanderi* (Foerster, 1850) *Temnothorax parvulus* (Schenck, 1852) *Temnothorax unifasciatus* (Latreille, 1798) *Tetramorium caespitum* (Linnaeus, 1758) *Tetramorium immigrans* Santschi, 1927

**Table 3:** Species richness, biodiversity indices, and similarity index of sites. For each Nonmetric Multidimensional Scaling (NMDS) cluster, mean values are listed (StdDataset).

NMDS Cluster	Site	Altitude (m a.s.l.)	Most Frequent Species and % of Occupied Baits	N Kits	N Baits	Species Richness	Simpson Diversity Index (1-D)	Shannon Diversity Index (H’)	Species Equitability Index (EH)	Sørensen–Dice Similarity Index (Average)
**Cluster 1**	Add	95	*T. immigrans.* (43%)	6	48	7	0.76	1.67	0.86	0.50
	Gro	215	*L. niger* (10%)	6	48	10	0.87	2.18	0.95	0.43
	NMi	140	*T. immigrans* (12%)	6	48	5	0.68	1.36	0.84	0.41
	LaCa	245	*L. niger/T.immigrans* (12%)	4	36	6	0.80	1.70	0.95	0.45
	Bre	175	*T. immigrans* (18%)	6	48	6	0.76	1.59	0.89	0.46
	Mean	174		5.6	45.6	6.8	0.77	1.70	0.90	0.45
**Cluster 2**	Ser	110	*M. rubra* (6%)	6	48	6	0.79	1.68	0.93	0.39
	LBin	20	*M. monomorium* (12%)	6	48	8	0.83	1.91	0.92	0.35
	CaBe	245	*M. sabuleti* (12%)	4	36	5	0.74	1.46	0.91	0.39
	Fon	30	*M. monomorium* (14%)	6	48	10	0.84	2.06	0.89	0.41
	Mean	101		5.5	45	7.2	0.80	1.78	0.91	0.38
**Cluster 3**	BCa	395	*L. emarginatus* (15%)	5	40	14	0.90	2.46	0.93	0.50
	BEr	845	*L. emarginatus* (10%)	5	40	9	0.87	2.11	0.96	0.43
	LaBr	380	*L. emarginatus/T. immigrans* (10%)	5	40	9	0.86	2.10	0.95	0.47
	VaM	340	*A. subterranea/T. unifasciatus* (6%)	6	48	11	0.89	2.29	0.96	0.41
	Mean	490		5.2	42	10.7	0.88	2.24	0.95	0.45

## Supplementary Materials

The following are available online at https://www.mdpi.com/2075-4450/11/10/678/s1, Table S1: List of the 12 parks of the A.R.E.A. Parchi network involved in the project, Table S2: Species checklist according to sites, Table S3: Values of Sørensen–Dice Similarity Index for all the possible comparisons between parks (StdDataset).
