# Supplementary material for: Public Engagement Provides First Insights on Po Plain Ant Communities and Reveals the Ubiquity of the Cryptic Species Tetramorium immigrans (Hymenoptera, Formicidae)
Source: Insects. 2020 Oct 7;11(10):678. doi: 10.3390/insects11100678 (PMC7601173; doi:10.3390/insects11100678)
Supplement: Supplementary file 1 [file insects-11-00678-s001.pdf]

Supplementary material to: Castracani, C.; Spotti, F.A.; Schifani, E.; Giannetti, D.; Ghizzoni, M.; Grasso, D.A.; Mori, A. Citizen science provides first insights on Po Plain ant communities and reveals the ubiquity of the cryptic invader *Tetramorium immigrans* (Hymenoptera, Formicidae)

Table S1. List of the 12 parks of the A.R.E.A. Parchi network involved in the project. The last column shows the number of sampled sites.

Park Name	Website address	Sites
Adda Nord	http://www.parcoaddanord.it/	1
Basso Brembo	http://www.parcobassobrembo.it/	1
Bosco Fontana	https://www.carabinieri.it/arma/oggi/organizzazione/organizzazione-per-la-tutela-forestale-ambientale-e-agroalimentare/utcb-e-le-130-riserve-naturali/utcb-di-verona/bosco-fontana	1
Campo dei Fiori	http://www.parcocampodeifiori.it/	1
Groane	http://www.parcogroane.it/	1
Monte Barro	http://www.parcobarro.lombardia.it/_parco/	2
Monte Canto e Bedesco	http://www.comitatomontecanto.it/	1
Nord Milano	https://parconord.milano.it/	1
Oglio Sud - Le Bine	http://www.ogliosud.it/pagina.php?id=14	1
Serio	https://www.parcodelserio.it/	1
Valle Lambro	https://www.parcovallelambro.it/	2
Valpredina-Misma	https://www.valpredina.eu/	1

Table S2. Species checklist according to sites. For each species, the % of baits in which they were found is expressed (StdDataset). A cross marks the species only present in the FDataset. Colors correspond to the clusters found in the NMDS analysis: orange = group 1, green = group 2 and red = group 3. Fiori Park is in white because it was excluded from StdDataset (see text for further details).

Specie	Add	Gro	NMi	LaCa	Bre	Ser	LBin	CaBe	Fon	BCa	BEr	LaBr	VaM	Fiori
T. subb.		2			+	2		3	2					
C. fallax	2								2					
C. later.										2				
C. lignip.											5			
F. clara							2							
F. cunic.	2	2		6	2	2	+		2	7		2	2	+
F. gagat.										7	2			
L. distin.		4												
L. emarg.	8	4	2				4			15	10	10	2	+
L. fulig.		+												
L. niger	4	10	4	12	8	+		3		2	5			+
L. paral.		4	+									2		
P. pygm.		4	+		8		2			2			+	
A. subter.				3					2	12	7	2	6	+
C. scutel.		2	+		+		4					2		
M. iberic.									2					
M. mon.							12		14					
M. hell.		+	+										2	
M. rubr.						6						2	4	
M. sabul.	2				+	2	10	12	4	2			+	
M. spec.				3	+					5	2		2	
P. pallid.										12			2	
S. fugax			6			2		3	2					
T. flavic.						+								
T. licht.					2					2			+	
T. nylan.													2	
T. parvul.												2		
T. unifas.		2		3	2		8		2	2	7		6	
T. caesp.	2		2			+	+			2	2	7	4	
T. immig.	43	4	12	12	18	4	10	9	8	10	5	10	4	+

Table S3. Values of Sørensen-Dice similarity index for all the possible comparisons between parks (StdDataset). Colors correspond to the clusters found in the NMDS analysis: orange = group 1, green = group 2 and red = group 3.

	Add	Gro	NMi	LaCa	Bre	Ser	LBin	CaBe	Fon	BCa	BEr	LaBr	VaM	Mean
**Add**	-	0.47	0.66	0.46	0.57	0.44	0.40	0.50	0.47	0.57	0.50	0.50	0.46	0.50
**Gro**	0.47	-	0.40	0.47	0.59	0.37	0.55	0.40	0.40	0.50	0.42	0.53	0.38	0.43
**NMi**	0.66	0.40	-	0.36	0.50	0.36	0.31	0.60	0.27	0.42	0.40	0.28	0.35	0.41
**LaCa**	0.46	0.47	0.36	-	0.46	0.33	0.28	0.36	0.50	0.60	0.67	0.40	0.55	0.45
**Bre**	0.57	0.59	0.50	0.46	-	0.31	0.40	0.33	0.35	0.66	0.50	0.37	0.44	0.46
**Ser**	0.44	0.37	0.36	0.33	0.31	-	0.28	0.73	0.67	0.30	0.13	0.40	0.33	0.39
**LBin**	0.40	0.55	0.31	0.28	0.40	0.28	-	0.15	0.44	0.45	0.35	0.35	0.31	0.35
**CaBe**	0.50	0.40	0.60	0.36	0.33	0.73	0.15	-	0.53	0.31	0.28	0.14	0.12	0.39
**Fon**	0.47	0.40	0.27	0.50	0.35	0.67	0.44	0.53	-	0.42	0.31	0.31	0.38	0.41
**BCa**	0.57	0.50	0.42	0.60	0.66	0.30	0.36	0.31	0.42	-	0.69	0.43	0.64	0.50
**BEr**	0.50	0.42	0.40	0.67	0.50	0.13	0.35	0.28	0.31	0.69	-	0.44	0.50	0.43
**LaBr**	0.50	0.53	0.28	0.40	0.37	0.40	0.35	0.14	0.31	0.43	0.44	-	0.50	0.47
**VaM**	0.46	0.38	0.35	0.55	0.44	0.33	0.31	0.12	0.38	0.64	0.50	0.50	-	0.41
